# Subaerial crust emergence hindered by phase-driven lower crust densification on early Earth

**DOI:** 10.1126/sciadv.adq1952

**Published:** 2024-09-11

**Authors:** Ming Tang, Hao Chen, Cin-Ty A. Lee, Wenrong Cao

**Affiliations:** ^1^ Key Laboratory of Orogenic Belt and Crustal Evolution, MOE, School of Earth and Space Sciences, Peking University, Beijing 100871, China.; ^2^ Department of Earth, Environmental and Planetary Sciences, Rice University, Houston, TX 77005, USA.; ^3^ Department of Geological Sciences and Engineering, University of Nevada, Reno, MS-172, 1664 N. Virginia St., Reno, NV 89557, USA.

## Abstract

Earth owes much of its dynamic surface to its bimodal hypsometry, manifested by high-riding continents and low-riding ocean basins. The thickness of the crust in the lithosphere exerts the dominant control on the long-wavelength elevations of continents. However, there is a limit to how high elevations can rise by crustal thickening. With continuous crustal thickening, the mafic lower crust eventually undergoes a densifying phase transition, arresting further elevation gain—an effect clearly observed in modern orogenic belts. On early Earth, lower crust densification should also limit how high a thickening crust can rise, regardless of the thickening mechanisms. We suggest that lower crust densification combined with a thicker oceanic crust in the Archean may have limited the whole-Earth topographic relief to 3 to 5 kilometers at most—half that of the present day. Unless the oceans were far less voluminous, limited relief would inevitably lead to a water world on early Earth.

## Teaser

Dense roots beneath the continents may have inhibited land emergence on early Earth.

## INTRODUCTION

Recent studies suggest that the area of exposed landmass in the first 1.5 to 2 billion years of Earth’s history was substantially smaller than today. For example, Archean volcanism recorded in greenstone belts appears to be dominated by submarine eruptions (*1*, *2*). The oxygen-18 isotopic signature of the Archean seawater recorded in hydrothermally altered oceanic crust also implies limited subaerial weathering before 3 billion years ago (*3*). Similarly, triple oxygen isotope compositions of shales point to a rapid emergence of subaerial crust near the Archean-Proterozoic boundary (*4*). While deep-time records have substantial uncertainties, these independent records exhibit similar patterns and raise the question of why subaerial environments were so scarce on early Earth.

The emergence of subaerial crust depends on seawater depth and whole-Earth topographic relief. Earth’s long-wavelength topography reflects combined dynamic and isostatic effects (*5*). Dynamic topography results from stresses associated with mantle flow, which generate transient topographic signals of <2 km (*6*, *7*). Isostatic topography is controlled by lateral variations in density and lithospheric thickness above a mantle compensation depth. Because the densities of oceanic and continental crust are notably less [by 300 to 600 kg/m^3^ (*8*)] than the ultramafic mantle, variations in crustal thickness have a large effect on elevation. The broadly positive correlation between long-wavelength elevation and crustal thickness of the continents indicates that the dominant control on elevation is the thickness of low-density crust (*6*, *9*). That is, to first order, ocean basins ride low because they are underlain by <10-km-thick basaltic crust, while mountain belts up to 8 km higher than the seafloor are underlain by 60- to 80-km-thick continental crust (*9*). These basic observations show that the formation of broad and sustained regions of high elevations requires crustal thickening either through magmatic or tectonic processes.

As topographic relief increases, two processes become important—erosion and gravitational collapse through lower crustal flow. Erosion and gravitational collapse have been recognized as critical processes limiting relief (*10*–*12*) and may explain the paucity of subaerial crust on early Earth if these processes were more efficient then (*13*, *14*). The efficiency of lower crustal flow is controlled by lateral pressure gradients induced by topography and by the viscosity of the lower crust, which, in turn, is controlled by crustal thermal state (*10*, *11*). The efficiency of erosion is influenced by climate, lithology, and topography (*15*). Therefore, both erosion and gravitational collapse operate as negative feedbacks to crustal thickening; elevations are controlled by the competition between tectonic forcing and these negative feedbacks (*16*). As long as tectonic forcing is high and/or negative feedbacks are weak, steady-state elevations can continue to rise. However, here, we show that internal processes, such as phase changes, ultimately define a firm upper bound on elevation.

## RESULTS AND DISCUSSION

### Elevation limited by lower crust densification

**Fig. 1.**
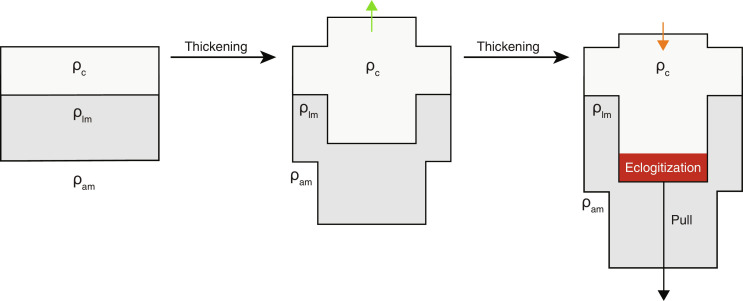
Airy isostasy and the effect of lower crust eclogitization/densification on elevation (cartoon not to scale). ρ_am_, asthenospheric mantle density; ρ_lm_, lithospheric mantle density; ρ_c_, crust density. ρ_am_ ≈ ρ_lm_ > ρ_c_.

Although crustal thickening is critical to increasing elevation, there is a limit to elevation gain by crustal thickening. If the crust exceeds a threshold thickness, then pressures become high enough to stabilize garnet, which, because of its high density, generates a dense lower crustal root (Fig. 1). This phase transformation is often referred to as lower crust eclogitization (*17*, *18*). As garnet mode increases, the density of the garnet-bearing lower crust can eventually surpass that of the mantle and result in negative buoyancy. Any further crustal thickening would only thicken the dense root, suppressing elevation gain and eventually driving subsidence (Fig. 1 and fig. S1) (*19*).

**Fig. 2.**
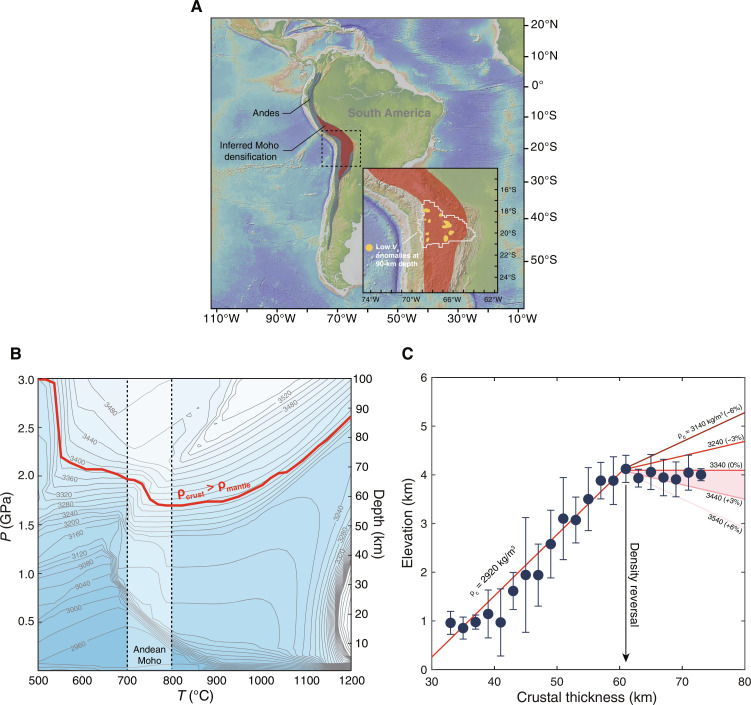
Two-stage evolution of elevation in the Andes. (**A**) Map showing the Andes and the areas of Moho densification inferred from isostasy calculations (crustal thickness > 60 km). The inset shows low *V*p anomalies in the shallow upper mantle beneath the central Andes, which was interpreted to reflect the upwelling of the asthenospheric mantle after lithosphere delamination (*32*). (**B**) Densities of average Andean arc basalts (water saturated, melt-free) plotted as contours with the numbers denoting densities in kilograms per cubic meter. The red curve marks the pressure above which the densities of Andean basalt exceed those of peridotite under the same pressure and temperature conditions. (**C**) Elevation of the Andes as a function of crustal thickness. The data are binned into 2-km crustal thickness intervals and are plotted as medians with median absolute deviations. The data were extracted from CRUST1.0 (fig. S7) (*77*). The red lines are the calculated correlations between elevation and crustal thickness. In our simplified isostasy model for the Andes, the influence of the lithospheric mantle was ignored as it is probably secondary (see texts for discussion). In CRUST1.0, crustal thickness is obtained from 1° averages of compiled active source seismic studies and receiver function studies published recently. Because the seismic properties of eclogites are hardly discernible from those of peridotites (*78*), the petrologic Moho can be deeper than the seismic Moho at crustal thickness > 60 km.

The Andean crust in South America is an example of orogenic thickening caused by compression and magmatism associated with the subduction of the Nazca Plate beneath South America (*20*). Variations in the relative magnitudes of compression, magmatism, erosion, and regional extension result in a wide range of crustal thicknesses along the length of the arc, making the Andes an ideal region to evaluate the influence of eclogitization on elevations (Fig. 2A).

Full isostatic analysis requires integration of all mass above a deep mantle compensation depth, such that the thicknesses and densities of all crustal and mantle (lithospheric and asthenospheric) layers are known. The density contrast between the lithospheric mantle and asthenospheric mantle is over an order of magnitude smaller than that between the crust and asthenospheric mantle (*8*, *21*). Thus, while thermal contraction and thickening of the lithospheric mantle beneath ocean basins with time play a key role in the subsidence of ocean basins, the elevation difference between ocean basins and continents is still strongly dependent on the difference in crustal thickness. Of interest to us is what controls isostatic elevation during active orogenic thickening before a thick thermal boundary layer has developed. In such a scenario, the lithospheric mantle is thin and plays a negligible role compared to that of the crust. For example, beneath Phanerozoic continents, the lithospheric mantle is thin [<100 km (*22*)], particularly in subduction zones where seismic velocities are generally low throughout the mantle wedge (*23*, *24*). The subordinate influence of the lithospheric mantle is further reflected by the lack of correlation between elevation and lithospheric mantle thickness in most continental areas (fig. S2). For these reasons, we consider crustal thickness and density variations to be the dominant control on isostatic elevation.

We first use phase equilibria modeling to calculate the densities of the Andean basaltic crust under various pressure and temperature conditions (see Materials and Methods). We find that at 700 to 800°C, a temperature range relevant to the average lower crust of the Andes (*25*), the average Andean basaltic crust becomes denser than the underlying peridotitic mantle between 1.8 and 1.9 GPa, or at ~60-km depth (Fig. 2B). The elevation–crustal thickness correlation of the Andes clearly shows a kink at ~60 km (Fig. 2C). For the crust less than 60 km thick, surface elevation and crustal thickness are positively correlated and roughly fit a bulk crustal density contour of ~2920 kg/m^3^, similar to previous density estimates (*26*). When crustal thickness surpasses ~60 km, the elevation–crustal thickness correlation shifts away and falls between the density contours of 3340 and 3440 kg/m^3^, equivalent to the crust being 0 to 3% denser than the mantle. This is also consistent with the modeled densities between 60 and 70 km (Fig. 2B).

The negatively buoyant crustal layer may be prone to detaching from the overlying crust and sinking into the mantle—a hypothetical process known as delamination or foundering (*18*, *27*–*30*). The high-density trend in Fig. 2C corresponds to the central Andes (Fig. 2A), where there is circumstantial evidence for lower crust eclogitization and delamination (*25*). Kay and coworkers (*29*, *31*) proposed lower crust eclogitization and delamination in this area based on the temporal evolution of magmatic activity and chemistry. These inferences were later supported by seismic tomography observations (*32*, *33*). Periodic surface uplift revealed by paleo-altitude reconstruction is also consistent with recent crustal thickening and lower crust removal events beneath the central Andean plateau (*34*).

The observed lack of elevation gain for crustal thickness > 60 km (Fig. 2C) indicates that elevation cannot increase indefinitely with crustal thickening, and in the case of the Andes, this dense root may not have delaminated in some places. If this eclogitized root detaches, then elevation will rise back up (*34*) until it reaches the inflection point defined by the onset of density reversal at the Moho.

Elevation suppression due to lower crust eclogitization has also been observed elsewhere. For example, the elevation-crustal thickness relationship in Tibet shows a similar pattern, albeit with a larger data scatter below 60 km crustal thickness (fig. S8). This larger data scatter may, in part, result from limited data at crustal thickness < 60 km in Tibet or complex variations in the density structure beneath Tibet due to Indian lithosphere underthrusting (*35*). The southern Ural Mountains in central Russia have an unusually low elevation of ~1 km, considering its >55-km-thick crust. This low elevation was also attributed to the presence of a thick eclogitic root, as confirmed by the active-source seismic reflection profile (*36*, *37*).

The kinked elevation–crustal thickness trend in the Andes (Fig. 2C) suggests that once a threshold in crustal thickness is attained (~60 km), eclogitization of the lower crust likely plays a dominant role in controlling elevation because erosion and gravitational collapse would preserve a positive correlation between crustal thickness and elevation (fig. S1). In orogens with crustal thicknesses below this eclogitization threshold, the coupling between erosion, gravitational collapse, and orogenesis determines steady-state mountain elevations.

### Low topographic relief on early Earth

Here, we explore how the above concepts may have manifested in the Archean and evaluate their implications for the lack of subaerial crust in Earth’s early history. Extrapolating Phanerozoic observations and concepts into the Archean is a bold endeavor. Continents as we know them today may not have even existed in the early Archean. It is also not clear if plate tectonics of the Phanerozoic was operating (*38*–*41*).

However, despite how different the Archean may have been, the importance of crustal thickness and phase transitions is universal. Both magmatic and orogenic thickening, perhaps in unique ways, clearly operated and formed the Archean crusts (*42*, *43*). We thus ask the question of what was the maximum whole-Earth relief in the Archean?

**Fig. 3.**
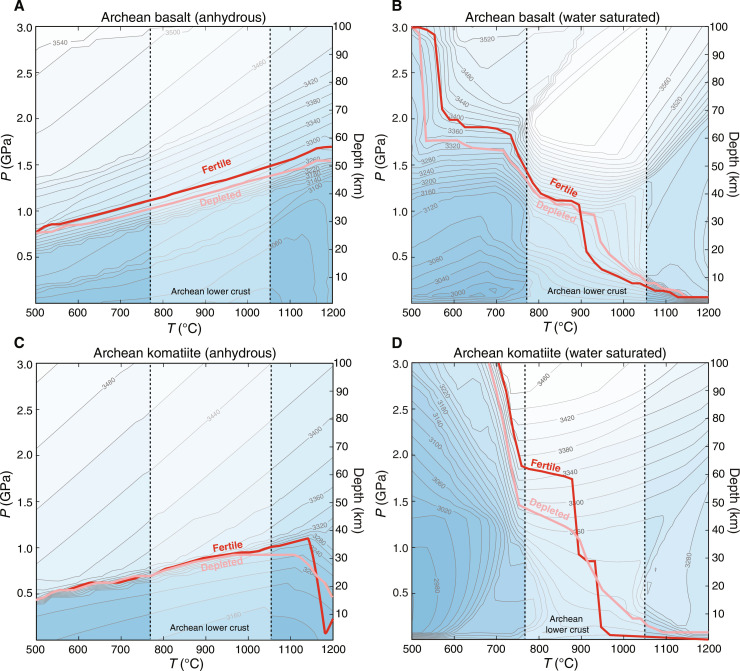
Densities of average Archean basalt and komatiite (melt-free). The red and pink curves mark the pressures above which the densities of Archean basalt/komatiite exceed those of fertile and depleted peridotites, respectively. (**A**) Average Archean basalt under anhydrous conditions. (**B**) Average Archean basalt under water-saturated conditions. (**C**) Average Archean komatiite under anhydrous conditions. (**D**) Average Archean komatiite under water-saturated conditions.

We now evaluate the depth of eclogitization for the Archean crust. We consider two endmember Moho compositions—basaltic and komatiitic—and two endmember peridotitic mantle compositions—fertile (same as the Andean mantle) and depleted. We model phase equilibria for average Archean basalt and komatiite under anhydrous and hydrous conditions (water saturated). Given the higher abundances of radioactive isotopes and hotter mantle in the Archean, the Moho temperature of a thickened Archean crust was probably higher than that of the Andean crust. The compilation of lower crustal temperatures by Brown and Johnson (*44*) shows that Archean deep crustal rocks (metamorphic P > 1 GPa) equilibrated at 777° to 1050°C. For simplicity, we take this temperature range as that of the Moho of the Archean thickened crust. This is a reasonable assumption because a thickened crust in the Archean is unlikely to have been colder than its modern counterparts such as the Andes. Within this temperature range, anhydrous Archean basalt and komatiite become denser than the depleted mantle at pressures of 0.9 to 1.4 GPa at most and fertile mantle at 1.0 to 1.5 GPa; under hydrous conditions, the pressure at which there is a density reversal increases to 1.3 to 1.4 GPa for depleted mantle and 1.4 to 1.8 GPa for fertile mantle (Fig. 3). Note that because of the large uncertainty in thermal state of the Archean deep crust, these density reversals could take place at even lower pressures than shown here, especially for hydrous crust conditions (Fig. 3, B and D). Density reversal depths, in general, are shallower in the Archean than in the Phanerozoic.

We are now faced with a dilemma for generating large whole-Earth topographic relief in the Archean. On one hand, the relatively shallow eclogitization suggests that the Archean crust would quickly lose its positive buoyancy as it thickens. On the other hand, Earth’s mantle is thought to have been hotter in the past (*45*–*49*). Although well-preserved records of Archean oceanic crust are limited (*50*–*53*), all other variables being similar, a hotter mantle might be expected to melt more extensively, thereby generating a thicker oceanic crust, perhaps reaching 25 to 35 km in the Archean based on mantle melting modeling (*45*, *54*). For a crust to ride high above the seafloor, it needs to be much thicker than the modern continental crust, and yet, negative buoyancy triggered by eclogitization would have limited the effect of crustal thickness on elevation gain.

**Fig. 4.**
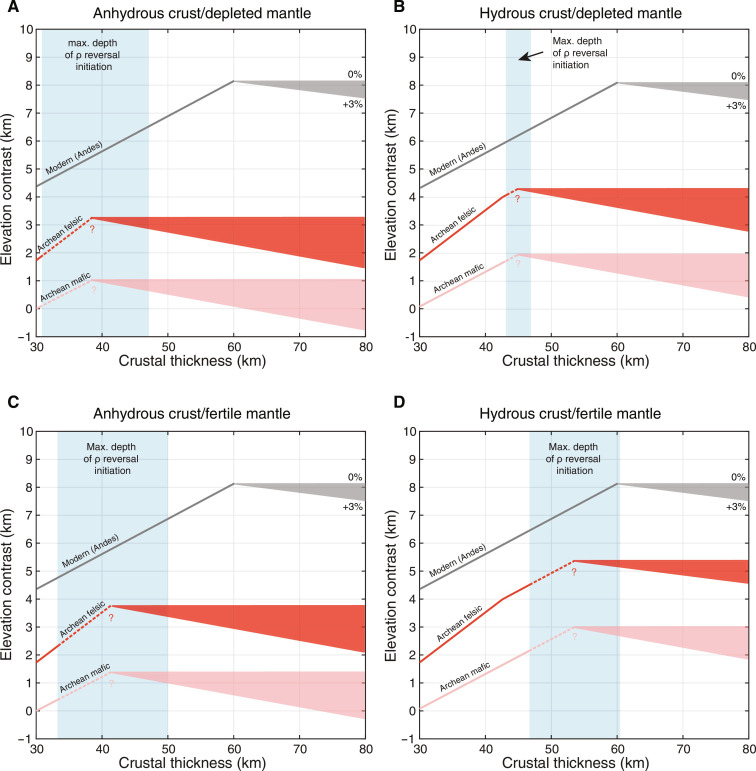
Elevation contrast between a thickened crust and seafloor in the Archean. In our calculation, we consider various crust and mantle conditions, including anhydrous crust on top of depleted mantle (**A**), hydrous (water-saturated) crust on top of depleted mantle (**B**), anhydrous crust on top of fertile mantle (**C**), and hydrous crust on top of fertile mantle (**D**). We assume an oceanic crust thickness of 30 km in the Archean (*45*). A seawater depth of 4 km (modern value) was used in all elevation calculations as a reference scenario. A deeper ocean would make the emergence of subaerial crust in the Archean even less likely. The blue bands denote the maximum depth intervals where mafic/ultramafic crust density begins to surpass mantle density based on Fig. 3. After the density reversal, the lower crust becomes 0 to 3% denser than the mantle, and the elevation contrasts evolve along the paths shown by the triangular envelopes. The question marks and dashed trajectory lines reflect the uncertainties in elevation inflection. The Andean paths are also shown for comparison.

As a proof of concept, we calculate the elevation contrast between the Archean thickened crust (protocontinents?) and seafloor. For the thickened crust, a dense crustal root forms once the Moho depth exceeds that of density reversal. For the crustal column shallower than the density reversal depth, we consider felsic and mafic compositions as both scenarios have been proposed for the Archean (*55*–*57*). With a ~30-km-thick oceanic crust, the elevation contrasts formed in the Archean are systematically lower than those of the present day (Fig. 4). An anhydrous crust (Fig. 4, A and C) rides slightly lower than a hydrous crust (Fig. 4, B and D) because density reversal may occur at slightly greater depths under water saturated conditions (Fig. 3). A mafic crust could rise only 1 to 3 km above the Archean seafloor. A felsic crust could rise higher due to its lower density. But even with a thick felsic crust, the maximum elevation contrasts may have been 3 to 5 km in the Archean as opposed to >8 km today (Fig. 4).

For a crust to rise >4 km higher than the seafloor in the Archean, the crust would have to be felsic, hydrous, and thickened to ~45 km (Fig. 4, B and D). A thick felsic crust in the Archean would be inevitably hot because of its high concentrations of heat-producing elements compared with those of a mafic crust (*16*, *58*). For example, a 45-km-thick crustal column composed of tonalite–trondhjemite–granodiorite (TTG) can reach >850°C at the Moho even in the absence of mantle magmatism (fig. S9). A hot hydrous crust scenario would lead to two problems. First, a hot hydrous crust would be rheologically weak (*16*, *58*). Under >850°C and hydrous conditions, the deep portion of the felsic crust could be largely molten and collapse almost instantly. Second, because the density reversal depths of hydrous mafic rocks negatively correlate with temperature and decrease to <40 km at >900°C (Fig. 3, B and D), a dense root would form at a notably shallower depth than indicated by the maximum intervals in Fig. 4 (B and D) and prevent the crust from rising further. Therefore, even a felsic crust would have difficulty rising >4 km above the Archean seafloor.

The higher thermal states in the Archean might have resulted in unusual crustal processes, such as widespread gravitational collapse or diapirism, which have also been invoked to infer low topographic relief on early Earth (*13*, *14*, *59*). Undoubtedly, all of these processes may have played a role in limiting the area of subaerial crust on early Earth. In addition, these processes also raise the question of whether the crust could be thickened to the point of lower crust eclogitization on early Earth, in which case elevation would be determined by steady-state crustal thickness.

As discussed above, the steady-state crustal thickness reflects the coupling between the processes that drive crustal thickening (orogenesis) and thinning (e.g., erosion and gravitational collapse) (*16*). If crustal thickening was far less efficient than crustal thinning processes in the Archean, then no crust may have reached the thickness threshold of eclogitization. In this case, Earth’s surface would form even less elevation contrasts as long as isostasy was at play. This scenario also appears to be inconsistent with the distinctively sharp, flat Moho beneath almost all Archean terrains undisturbed by younger orogenic events (*60*–*62*). The formation of these sharp Mohos is generally attributed to the delamination of garnet-bearing dense roots (*60*–*62*). This would indicate that the growth of dense crustal roots was a widespread process in the formation of Archean cratons.

Our analysis does not indicate lower crust eclogitization as the sole factor in limiting elevation contrast on early Earth. However, regardless of the various elevation-limiting processes, isostasy and lower crust eclogitization set an upper limit on elevation contrast. The maximum elevation contrast calculated for the Archean Earth, whether or not it was actually achieved, is already sufficiently low to inhibit the emergence of subaerial crust—for a crust to rise ~4 km (average modern seawater depth) above the ocean floor in the Archean, that crust may have already reached the threshold thickness of lower crust eclogitization (Fig. 4).

In the above analysis, we did not consider the contribution from the lithospheric mantle which would form as the mantle cools. As discussed earlier, the effect of the lithospheric mantle is secondary compared to that of the crust if their thicknesses are comparable. Archean cratons, however, are underlain by unusually thick lithospheric mantle keels (*22*). These mantle keels are composed of melt-depleted and hence low-density residual peridotites, but because the cratonic mantle is also cold, this compositional buoyancy would be lessened by a negative thermal buoyancy (*63*). It may even be possible that these mantle keels are negatively buoyant due to the presence of dense pyroxenite lithologies in the lithosphere that are rarely accounted for in petrologic models. A net negative buoyancy of the cratonic lithospheric mantle is also supported by more recent global topography and geoid observations (*64*, *65*). In Earth’s early history, the hotter ambient mantle may have enhanced this negative buoyancy of the bulk cratonic lithosphere (*9*), and the cratonic mantle keels may have been even thicker (*66*). Thus, although we cannot precisely quantify the influence of the lithospheric mantle, its effect would only be to further suppress whole-Earth topographic relief.

With a limited topographic relief, Earth’s surface would be flatter, and subaerial crust could only emerge if seawater depth was substantially lower. However, the available observations do not favor shallower oceans on early Earth. Seawater depth depends on ocean volume, the area of subaerial crusts, and the volume of ocean ridges, with ocean volume playing the dominant role (*48*). Although substantial uncertainties persist in current estimates, ocean volume appears to be larger on early Earth based on the hydrogen isotopes recorded in serpentinized Archean oceanic crust (*67*, *68*), mantle mineral physics constraint (*69*), and secular water cycling modeling results (*21*).

Collectively, our results suggest that early Earth was inevitably covered by a global ocean, no matter how tectonically or magmatically active Earth may have been in its early history. Only the highest peaks in active orogenic belts would have been above sea level, forming isolated islands in this ancient water world (Fig. 4). These islands may have been the sole sources of subaerial weathering and terrigenous sediment formations on early Earth, while the vast portions of Earth’s surface would be below sea level.

### Rapid expansion of land?

**Fig. 5.**
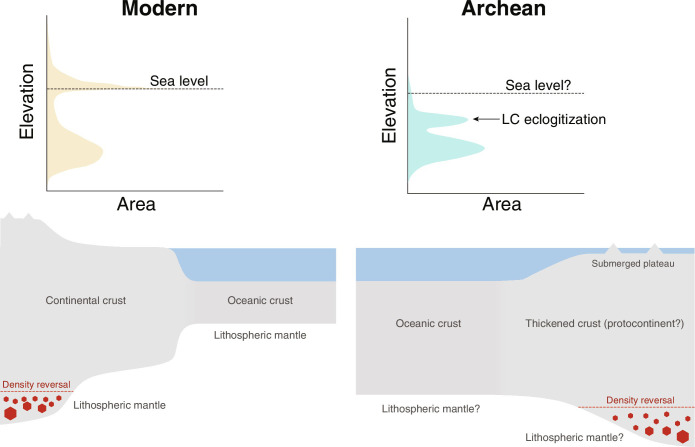
Conceptual diagrams and cartoons showing Earth’s hypsometry at present and in the Archean. In the conceptual hypsometry diagram for the Archean, the high elevation mode formed as a result of eclogitization beneath thickened crusts. Cartoon not to scale.

Despite the paucity of subaerial crust, Earth’s hypsometry may still have been bimodal in the Archean, and high elevations may have formed in areas of thickened crusts where the roots were eclogitized (Fig. 5). Crustal thickening may have nucleated at oceanic plateaus driven by intense plume magmatism (*39*) or by orogenesis (*16*, *39*, *70*). In either case, the crusts, once thickened to the threshold of eclogitization at the Moho, would attain similar elevations and remain submerged (Figs. 4 and 5). As the ambient mantle temperature dropped and the oceanic crust thinned, these submerged plateaus may have risen to sea level and formed the first subaerial surfaces. Because of the near-uniform elevations of these plateaus, the expansion of subaerial landmasses may have been extremely fast once these plateaus reached sea level. The rapid expansion of exposed landmasses may have had profound influences on Earth’s surface environment as hydrologic and weathering cycles were quickly reshaped (*4*).

## MATERIALS AND METHODS

### Phase equilibria modeling

We calculated the densities of average Andean basalt, Archean basalt, Archean komatiite, depleted peridotite, and fertile peridotite using phase equilibria modeling, which was done using the software Perple_X (version 6.9.1) (*71*) in the Na_2_O-CaO-K_2_O-FeO-MgO-Al_2_O_3_-SiO_2_-H_2_O-TiO_2_-O_2_ (NCKFMASHTO) system with the hp633ver database (*72*). The major element compositions of these rock endmembers are listed in Table 1. We assumed Fe^3+^/∑Fe =0.1 for all simulations. Solution models used in the calculations are as follows: melt [melt (HGP)] (*73*), olivine [O(HGP)] (*73*), orthopyroxene [Opx(HGP)] (*73*), clinopyroxene [Cpx(HGP)] (*73*), plagioclase [Fsp(C1)] (*74*), spinel [Sp(HGP)] (*73*), garnet [Gt(HGP)] (*73*), and ilmenite [Ilm(WPH)] (*75*) for peridotitic systems; melt [melt(G)] (*76*), olivine [O(HP)] (*72*), amphibole [cAmph(G)] (*76*), orthopyroxene [Opx(W)] (*75*), clinopyroxene [Augite(G)] (*76*), plagioclase [Fsp(C1)] (*75*), garnet [Gt(W)] (*75*), and ilmenite [Ilm(WPH)] (*75*) for komatiitic systems; melt [melt(G)] (*76*), amphibole [cAmph(G)] (*76*), orthopyroxene [Opx(W)] (*75*), clinopyroxene [Augite(G)] (*76*), plagioclase [Fsp(C1)] (*74*), garnet [Gt(W)] (*75*), ilmenite [Ilm(WPH)] (*75*), mica [Mica(W)] (*75*), and biotite [Bi(W)] (*75*) for basaltic systems. Quartz, rutile, titanite, and water were considered pure phases. The *P*-*T* phase diagrams and equilibrium mineral assemblages were calculated at discrete *P*-*T* points for every 18°C and 0.08 GPa from 500° to 1200°C and 0 to 3 GPa (figs. S3 to S6 and data S1). The densities were then obtained from the modeled equilibrium mineral assemblages on a melt-free basis.

**Table 1.** Compositions used for phase equilibria modeling.

	Archean komatiite*	Archean basalt*	Andean basalt^†^	Fertile peridotite^‡^	Depleted peridotite^‡^
SiO_2_	46.7	49.2	48.3	45.0	43.3
TiO_2_	0.50	0.80	1.66	0.20	0.05
Al_2_O_3_	7.50	13.53	15.48	4.45	0.99
FeO	9.28	10.31	8.87	7.25	6.48
Fe_2_O_3_	1.15	1.27	1.10	0.89	0.80
CaO	8.73	10.40	9.52	3.55	0.7
MgO	21.78	9.49	8.73	37.8	46.19
MnO	0.18	0.28	0.16	0.135	0.14
K_2_O	0.26	0.52	1.21	0.029	0
Na_2_O	0.56	1.82	3.21	0.36	0
P_2_O_5_	0.04	0.09	0.43	0	0

*Average compositions calculated using the compilation from (*55*).

†Average compositions calculated using data from GeoRoc (https://georoc.eu/).

‡From (*79*).

### Isostasy calculation

Elevation was calculated on the basis of the Airy isostasy principle

$$\begin{array}{c} h_{\text{cc}}*\rho_{\text{cc}}*g+h_{\text{rt}}*\rho_{\text{rt}}*g+h_{\text{cw}}*\rho_{\text{sw}}*g=h_{\text{sw}}*\rho_{\text{sw}}*g\\ +h_{\text{oc}}*\rho_{\text{oc}}*g+h_{m}*\rho_{m}*g \end{array}$$ (1)

The left side of the equation calculates the total gravity of a thickened crust (or continental crust); the right side of the equation calculates the total gravity of the oceanic crust and mantle column above the compensation depth. ρ_cc_, ρ_rt_, ρ_oc_, ρ_sw_, and ρ_m_ represent the densities of the crustal portion shallower than the eclogitization depth, eclogitized crustal root, oceanic crust, seawater, and mantle, respectively; *h*_cc_, *h*_rt_, *h*_sw_, *h*_cw_, *h*_oc_, and *h*_m_ are the thicknesses of the crustal portion shallower than the eclogitization depth and eclogitized crustal root, depth of seawater above the seafloor, depth of seawater above the thickened crust (=0 after emergence), the thickness of the oceanic crust, and the height of the mantle column from the oceanic crust to the compensation depth, respectively (fig. S10); *g* is the acceleration due to gravity. We assumed a modern oceanic crust density of 3000 kg/m^3^. Adopting an average modern seawater depth of 4 km and a mantle density of 3340 kg/m^3^, we derived a bulk crust density of 2920 kg/m^3^ for the Andean crust of 30 to 60 km thickness based on the elevation and crustal thickness data from CRUST1.0. This bulk density probably reflects the composite density of granites + andesites + minor basalts and falls in the density range estimated by integrated three-dimensional density modeling using available geological and geophysical data (*26*).

While bulk crust density may vary from place to place, the remarkable correlation shown in Fig. 2C indicates that this density variation plays a secondary role compared with that of crustal thickness. It should also be pointed out that the bulk crust density from the model is linked to the assumed mantle density and seawater depth. A bulk crust density of 2920 kg/m^3^ represents the best fit to the CRUST1.0 data when assuming a mantle density of 3340 kg/m^3^ and an average seawater depth of 4 km. If we consider a wider density range of 2700 to 3000 kg/m^3^ for the crustal column, then the density reversal depth (inflection point in Fig. 2C) may vary from 60 to 70 km.

A dense crustal root forms as the Moho pressure surpasses that of the density reversal calculated by phase equilibria modeling (*h*_rt_ = 0 when *h*_cc_ < density reversal depth). We consider this root to be 0 to 3% denser than the mantle beneath based on the Andean trend.

Before the thickened crust emerges from seawater, elevation can be calculated as

$$H=-h_{\text{cw}}$$ (2)

After emergence,

$$H=h_{\text{cc}}+h_{\text{rt}}-h_{\text{sw}}-h_{\text{oc}}-h_{m}$$ (3)

The elevation contrast (*E*_c_)—elevation difference between the thickened crust and oceanic crust

$$E_{c}=h_{\text{cc}}+h_{\text{rt}}-h_{\text{oc}}-h_{m}$$ (4)

For the Archean isostasy calculations, we adopted a bulk density of 2920 kg/m^3^ for the thickened Archean felsic crust, the same as that of the Andean crust. For thickened Archean mafic crust, we first computed the average densities of Archean basalt between 0 and 1 GPa for each temperature modeled between 500° and 1200°C and used the minimum average densities as the bulk crust densities. This approach may underestimate the bulk densities of Archean mafic crust because the denser portions of the crusts at >1 GPa are ignored. As a consequence, the elevations of the Archean mafic crust may be overestimated. In doing so, we derived bulk densities of 3055 and 3049 kg/m^3^ for Archean anhydrous and hydrous mafic crusts, respectively. We also adopted the bulk density of the Archean anhydrous mafic crust (3055 kg/m^3^) as that of the Archean oceanic crust.

## Supplementary Materials

### The PDF file includes:

Figs. S1 to S10 Legends for data S1 and S2 References
