# Supplementary material for: Subaerial crust emergence hindered by phase-driven lower crust densification on early Earth
Source: Sci Adv. 2024 Sep 11;10(37):eadq1952. doi: 10.1126/sciadv.adq1952 (PMC11389787; doi:10.1126/sciadv.adq1952)
Supplement: Supplementary file 1 — Figs. S1 to S10 Legends for data S1 and S2 References [file sciadv.adq1952_sm.pdf]

Science Advances
AAAS

Supplementary Materials for

Subaerial crust emergence hindered by phase-driven lower crust densification on early Earth

Ming Tang *et al.*

Corresponding author: Ming Tang, mingtang@pku.edu.cn

Sci. Adv. **10**, eadq1952 (2024)
DOI: 10.1126/sciadv.adq1952

The PDF file includes:

Figs. S1 to S10
Legends for data S1 and S2
References

Other Supplementary Material for this manuscript includes the following:

Data S1 and S2

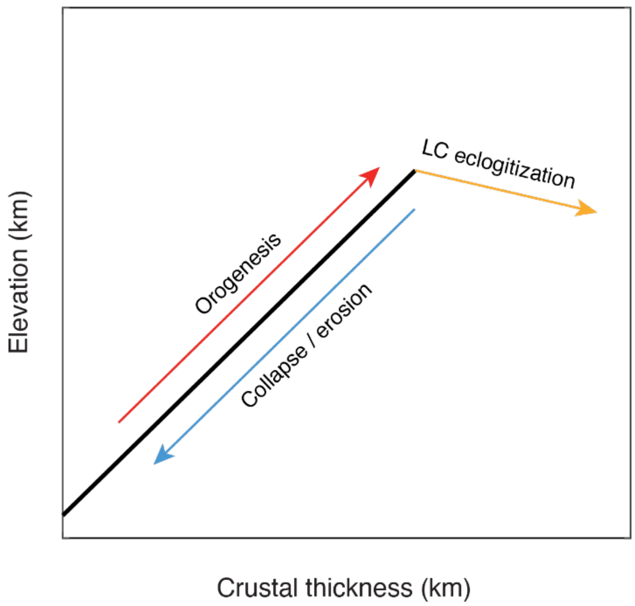

Fig. S1. Conceptual diagram showing the evolution of elevation for orogenesis, gravitational collapse, erosion, and lower crust eclogitization.

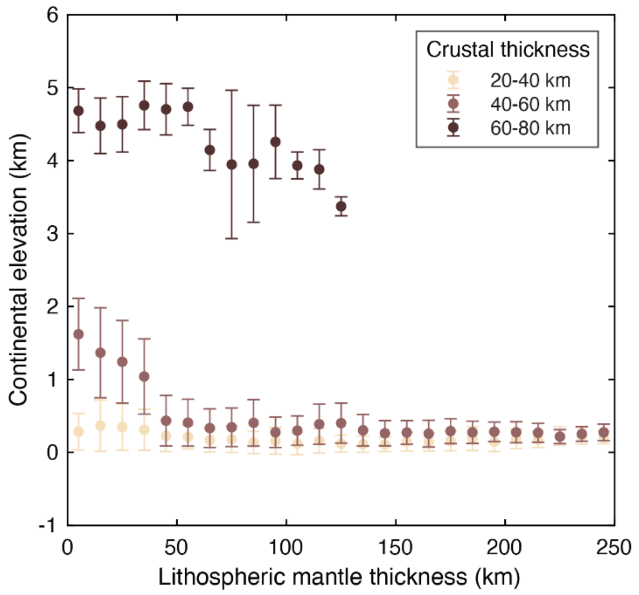

Fig. S2. Modern continental elevation vs. lithosphere thicknesses. The data are plotted as median with median absolute deviation for each 10 km lithospheric mantle thickness bin.

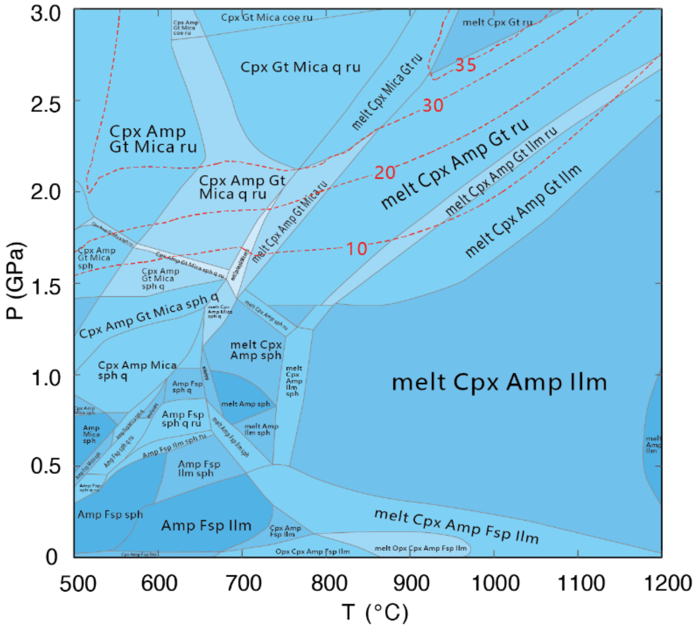

Fig. S3. Simplified P–T phase diagram for average Andean basalt under water saturated conditions. The red dashed lines mark the contours of garnet mode denoted by the numbers. The data used to calculate the average composition of Andean basalts are from GeoRoc (https://georoc.eu/).

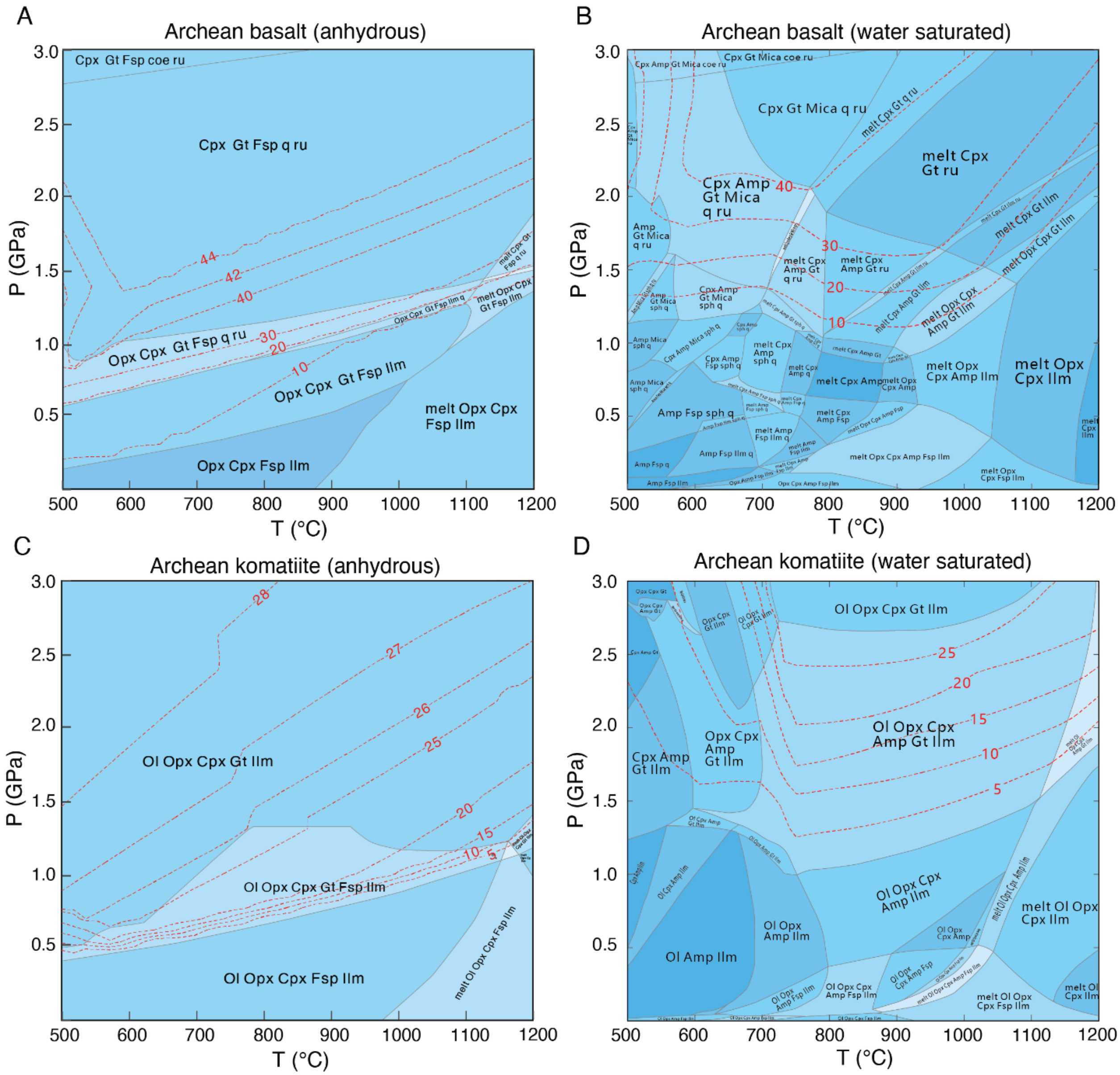

Fig. S4. Simplified P–T phase diagrams for average Archean basalt and komatiite under anhydrous and hydrous (water saturated) conditions. The red dashed lines mark the contours of garnet mode denoted by the numbers. The data used to calculate the average composition of Andean basalts are from ref. (*55*).

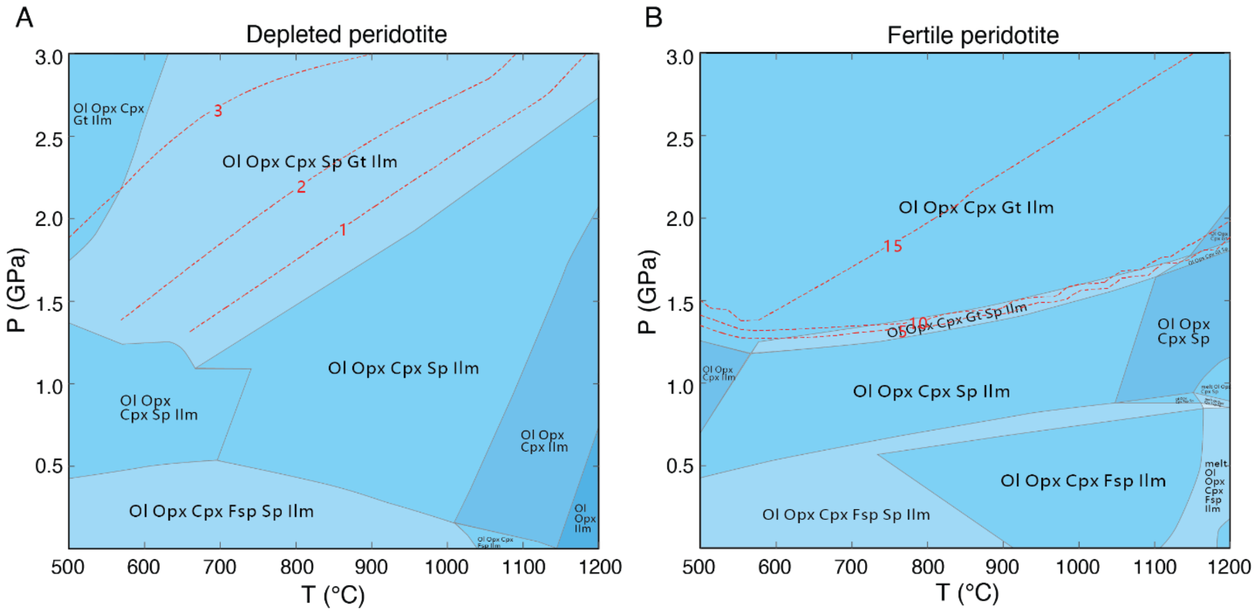

Fig. S5. Simplified P–T phase diagrams for depleted and fertile peridotites. The red dashed lines mark the contours of garnet mode denoted by the numbers. Fertile peridotite composition data are from ref. (*79*); depleted peridotite (cratonic peridotite) composition data are from ref. (*80*).

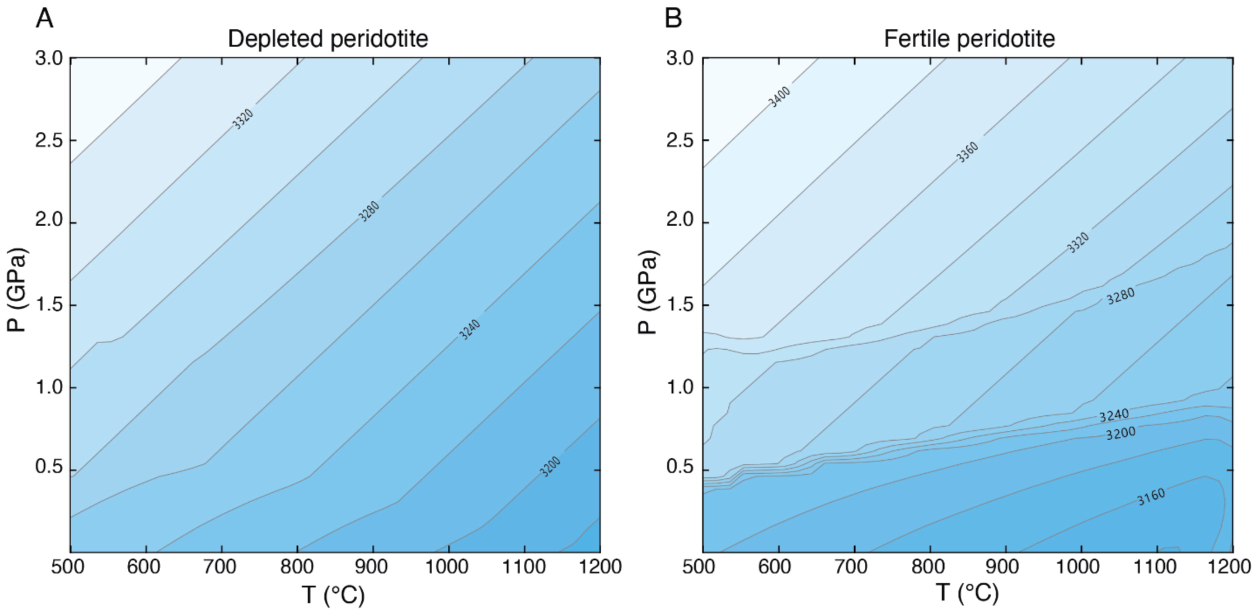

Fig. S6. Calculated densities of depleted and fertile peridotites.

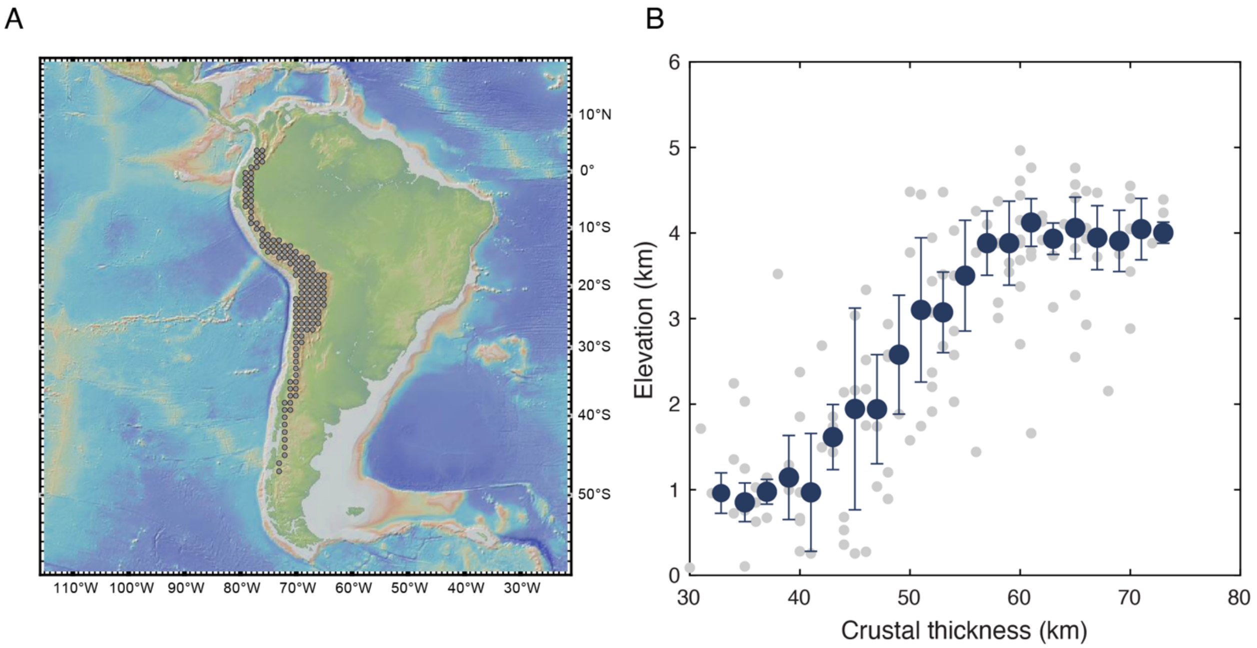

Fig. S7. Relationship between elevation and crustal thickness in the Andes. A. CRUST 1.0 grid data coverage in the Andes. **B.** Plot showing elevation and crustal thickness grid data and binned medians with median absolute deviations. Bin size = 2 km.

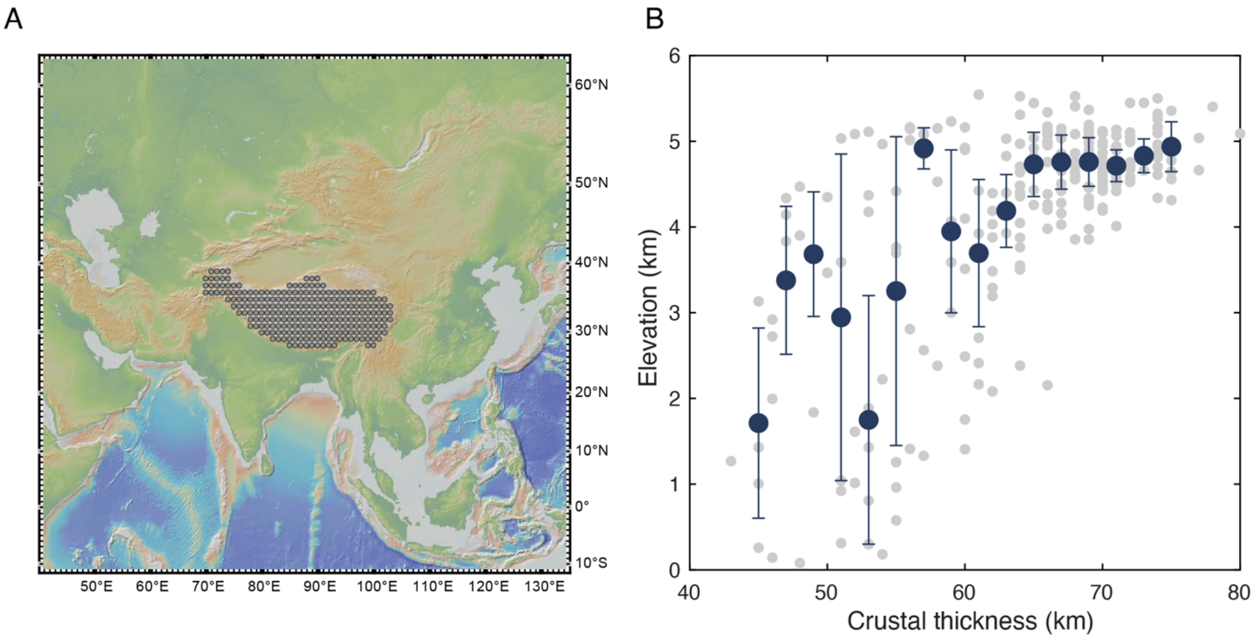

Fig. S8. Relationship between elevation and crustal thickness in Tibet. A. CRUST 1.0 grid data coverage in Tibet. **B.** Plot showing elevation and crustal thickness grid data and binned medians with median absolute deviations. Bin size = 2 km.

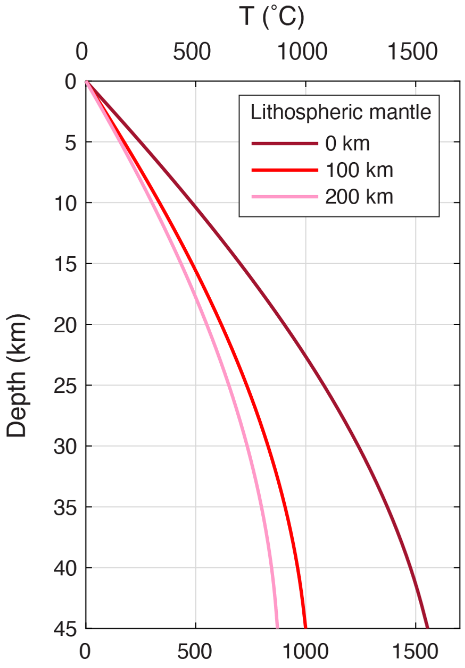

Fig. S9. Calculated steady state geothermal gradients for a 45 km thick felsic Archean crust in the absence of mantle magmatism. We assume that this felsic crust has the same bulk composition as the average Archean TTG (*81*). We consider three lithospheric mantle thicknesses. Our calculated geotherms appear to be slightly hotter than those proposed in ref. (*82*), which could be due to the slight differences in the U, Th, and K concentrations adopted for the TTG component.

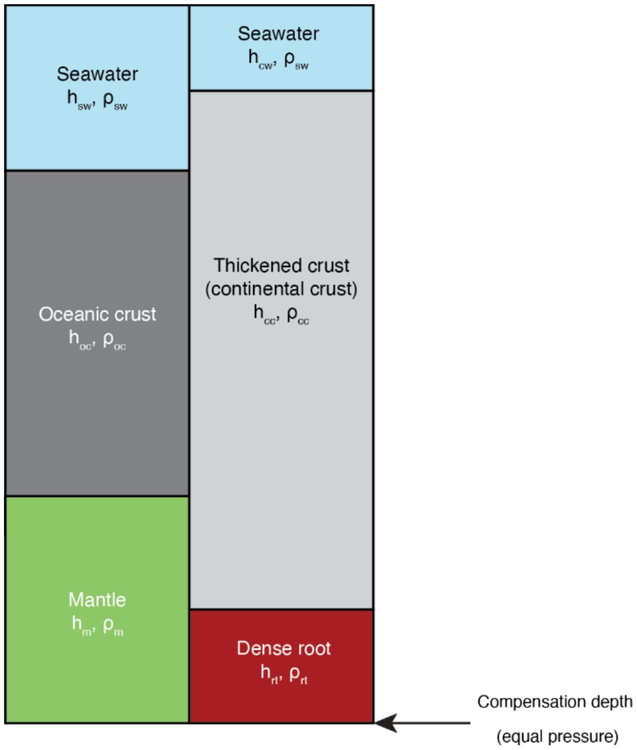

Fig. S10. Isostasy model set-up. Note that h_m can be negative in equation 1. This means that the mantle layer above the compensation depth is actually on the thickened crust (continental crust) side.

Data S1. (separate file)
Computed densities for peridotite, Archean komatiite, and Archean basalt.

Data S2. (separate file)
Crustal thickness and elevation data for the Andes and Tibet

REFERENCES AND NOTES

1. L. R. Kump, M. E. Barley, Increased subaerial volcanism and the rise of atmospheric oxygen 2.5 billion years ago. *Nature* **448**, 1033–1036 (2007).

2. C. T. Liu, Y. S. He, Rise of major subaerial landmasses about 3.0 to 2.7 billion years ago. *Geochem. Perspect. Lett.* **18**, 10.7185/geochemlet.2115 (2021).

3. B. W. Johnson, B. A. Wing, Limited Archaean continental emergence reflected in an early Archaean 18O-enriched ocean. *Nat. Geosci.* **13**, 243–248 (2020).

4. I. N. Bindeman, D. O. Zakharov, J. Palandri, N. D. Greber, N. Dauphas, G. J. Retallack, A. Hofmann, J. S. Lackey, A. Bekker, Rapid emergence of subaerial landmasses and onset of a modern hydrologic cycle 2.5 billion years ago. *Nature* **557**, 545–548 (2018).

5. A. M. Forte, D. B. Rowley, Earth's isostatic and dynamic topography—A critical perspective. *Geochem. Geophys. Geosyst.* **23**, e2021GC009740 (2022).

6. N. Flament, M. Gurnis, R. D. Müller, A review of observations and models of dynamic topography. *Lithosphere* **5**, 189–210 (2013).

7. J. Braun, The many surface expressions of mantle dynamics. *Nat. Geosci.* **3**, 825–833 (2010).

8. S. Lamb, J. D. Moore, M. Perez-Gussinye, T. Stern, Global whole lithosphere isostasy: Implications for surface elevations, structure, strength, and densities of the continental lithosphere. *Geochem. Geophys. Geosyst.* **21**, e2020GC009150 (2020).

9. C.-T. A. Lee, J. Caves, H. Jiang, W. Cao, A. Lenardic, N. R. McKenzie, O. Shorttle, Q.-z. Yin, B. Dyer, Deep mantle roots and continental emergence: Implications for whole-Earth elemental cycling, long-term climate, and the Cambrian explosion. *Int. Geol. Rev.* **60**, 431–448 (2018).

10. P. Rey, O. Vanderhaeghe, C. Teyssier, Gravitational collapse of the continental crust: Definition, regimes and modes. *Tectonophysics* **342**, 435–449 (2001).

11. M. K. Clark, L. H. Royden, Topographic ooze: Building the eastern margin of Tibet by lower crustal flow. *Geology* **28**, 703–706 (2000).

12. C.-T. A. Lee, S. Thurner, S. Paterson, W. Cao, The rise and fall of continental arcs: Interplays between magmatism, uplift, weathering, and climate. *Earth Planet. Sci. Lett.* **425**, 105–119 (2015).

13. P. F. Rey, N. Coltice, Neoarchean lithospheric strengthening and the coupling of Earth's geochemical reservoirs. *Geology* **36**, 635–638 (2008).

14. N. Flament, P. F. Rey, N. Coltice, G. Dromart, N. Olivier, Lower crustal flow kept Archean continental flood basalts at sea level. *Geology* **39**, 1159–1162 (2011).

15. K. X. Whipple, The influence of climate on the tectonic evolution of mountain belts. *Nat. Geosci.* **2**, 97–104 (2009).

16. M. Tang, C.-T. A. Lee, R. L. Rudnick, K. C. Condie, Rapid mantle convection drove massive crustal thickening in the late Archean. *Geochim. Cosmochim. Acta* **278**, 6–15 (2020).

17. H. Austrheim, Eclogitization of lower crustal granulites by fluid migration through shear zones. *Earth Planet. Sci. Lett.* **81**, 221–232 (1987).

18. C. T. A. Lee, in *Treatise on Geochemistry (Second Edition)*, H. D. Holland, K. K. Turekian, Eds. (Elsevier, 2014), pp. 423–456.

19. P. G. DeCelles, M. N. Ducea, P. Kapp, G. Zandt, Cyclicity in Cordilleran orogenic systems. *Nat. Geosci.* **2**, 251–257 (2009).

20. C.-T. A. Lee, L. Y. Yeung, N. R. McKenzie, Y. Yokoyama, K. Ozaki, A. Lenardic, Two-step rise of atmospheric oxygen linked to the growth of continents. *Nat. Geosci.* **9**, 417–424 (2016).

21. J. Korenaga, N. J. Planavsky, D. A. Evans, Global water cycle and the coevolution of the Earth's interior and surface environment. *Philos. Trans. A* **375**, 20150393 (2017).

22. I. M. Artemieva, W. D. Mooney, On the relations between cratonic lithosphere thickness, plate motions, and basal drag. *Tectonophysics* **358**, 211–231 (2002).

23. R. van der Hilst, Complex morphology of subducted lithosphere in the mantle beneath the Tonga trench. *Nature* **374**, 154–157 (1995).

24. S. Widiyantoro, R. van der Hilst, Structure and evolution of lithospheric slab beneath the Sunda Arc, Indonesia. *Science* **271**, 1566–1570 (1996).

25. S. L. Beck, G. Zandt, The nature of orogenic crust in the central Andes. *J. Geophys. Res.* **107**, ESE 7-1–ESE 7-16 (2002).

26. C. Meeßen, J. Sippel, M. Scheck-Wenderoth, C. Heine, M. Strecker, Crustal Structure of the Andean foreland in Northern Argentina: Results from data-integrative three-dimensional density modeling. *J. Geophys. Res. Solid Earth* **123**, 1875–1903 (2018).

27. M. N. Ducea, J. B. Saleeby, The age and origin of a thick mafic–ultramafic keel from beneath the Sierra Nevada batholith. *Contrib. Mineral. Petrol.* **133**, 169–185 (1998).

28. O. Jagoutz, M. D. Behn, Foundering of lower island-arc crust as an explanation for the origin of the continental Moho. *Nature* **504**, 131–134 (2013).

29. R. W. Kay, S. Mahlburg Kay, Delamination and delamination magmatism. *Tectonophysics* **219**, 177–189 (1993).

30. R. L. Rudnick, Making continental crust. *Nature* **378**, 571–578 (1995).

31. S. M. Kay, B. Coira, J. Viramonte, Young mafic back arc volcanic rocks as indicators of continental lithospheric delamination beneath the Argentine Puna Plateau, central Andes. *J. Geophys. Res. Solid Earth* **99**, 24323–24339 (1994).

32. S. C. Myers, S. Beck, G. Zandt, T. Wallace, Lithospheric-scale structure across the Bolivian Andes from tomographic images of velocity and attenuation for P and S waves. *J. Geophys. Res. Solid Earth* **103**, 21233–21252 (1998).

33. X. Liang, E. Sandvol, S. Kay, B. Heit, X. Yuan, P. Mulcahy, C. Chen, L. Brown, D. Comte, P. Alvarado, Delamination of southern Puna lithosphere revealed by body wave attenuation tomography. *J. Geophys. Res. Solid Earth* **119**, 549–566 (2014).

34. C. N. Garzione, G. D. Hoke, J. C. Libarkin, S. Withers, B. MacFadden, J. Eiler, P. Ghosh, A. Mulch, Rise of the Andes. *Science* **320**, 1304–1307 (2008).

35. T. J. Owens, G. Zandt, Implications of crustal property variations for models of Tibetan plateau evolution. *Nature* **387**, 37–43 (1997).

36. R. Carbonell, A. Pérez-Estaún, J. Gallart, J. Diaz, S. Kashubin, J. Mechie, R. Stadtlander, A. Schulze, J. Knapp, A. Morozov, Crustal root beneath the Urals: Wide-angle seismic evidence. *Science* **274**, 222–224 (1996).

37. J. Knapp, D. Steer, L. Brown, R. Berzin, A. Suleimanov, M. Stiller, E. Lüschen, D. Brown, R. Bulgakov, S. Kashubin, Lithosphere-scale seismic image of the southern Urals from explosion-source reflection profiling. *Science* **274**, 226–228 (1996).

38. P. A. Cawood, P. Chowdhury, J. A. Mulder, C. J. Hawkesworth, F. A. Capitanio, P. M. Gunawardana, O. Nebel, Secular evolution of continents and the Earth system. *Rev. Geophys.* **60**, e2022RG000789 (2022).

39. J. H. Bédard, Stagnant lids and mantle overturns: Implications for Archaean tectonics, magmagenesis, crustal growth, mantle evolution, and the start of plate tectonics. *Geosci. Front.* **9**, 19–49 (2018).

40. T. Kusky, B. F. Windley, A. Polat, L. Wang, W. Ning, Y. Zhong, Archean dome-and-basin style structures form during growth and death of intraoceanic and continental margin arcs in accretionary orogens. *Earth Sci. Rev.* **220**, 103725 (2021).

41. B. F. Windley, T. Kusky, A. Polat, Onset of plate tectonics by the Eoarchean. *Precambrian Res.* **352**, 105980 (2021).

42. P. Chowdhury, J. A. Mulder, P. A. Cawood, S. Bhattacharjee, S. Roy, A. N. Wainwright, O. Nebel, S. Mukherjee, Magmatic thickening of crust in non–plate tectonic settings initiated the subaerial rise of

Earth’s first continents 3.3 to 3.2 billion years ago. *Proc. Natl. Acad. Sci. U.S.A.* **118**, e2105746118 (2021).

43. B. F. Windley, A. A. Garde, Arc-generated blocks with crustal sections in the North Atlantic craton of West Greenland: Crustal growth in the Archean with modern analogues. *Earth Sci. Rev.* **93**, 1–30 (2009).

44. M. Brown, T. Johnson, Secular change in metamorphism and the onset of global plate tectonics. *Am. Mineral.* **103**, 181–196 (2018).

45. C. Herzberg, K. Condie, J. Korenaga, Thermal history of the Earth and its petrological expression. *Earth Planet. Sci. Lett.* **292**, 79–88 (2010).

46. C. Herzberg, R. Rudnick, Formation of cratonic lithosphere: An integrated thermal and petrological model. *Lithos* **149**, 4–15 (2012).

47. C.-T. A. Lee, E. J. Chin, Calculating melting temperatures and pressures of peridotite protoliths: Implications for the origin of cratonic mantle. *Earth Planet. Sci. Lett.* **403**, 273–286 (2014).

48. S. Galer, Interrelationships between continental freeboard, tectonics and mantle temperature. *Earth Planet. Sci. Lett.* **105**, 214–228 (1991).

49. M. J. Bickle, Implications of melting for stabilisation of the lithosphere and heat loss in the Archaean. *Earth Planet. Sci. Lett.* **80**, 314–324 (1986).

50. T. M. Kusky, *Precambrian Ophiolites and Related Rocks* (Elsevier, 2004).

51. W. Ning, T. Kusky, L. Wang, B. Huang, Archean eclogite-facies oceanic crust indicates modern-style plate tectonics. *Proc. Natl. Acad. Sci. U.S.A.* **119**, e2117529119 (2022).

52. W. Ning, T. Kusky, L. Wang, B. Huang, Reply to Zou et al.: Neoarchean eclogite-facies oceanic crust in the North China Craton. *Proc. Natl. Acad. Sci. U.S.A.* **119**, e2210169119 (2022).

53. Y. Zou, X. Chu, J. Wu, L. Zhao, No evidence for Archean eclogite-facies metamorphism. *Proc. Natl. Acad. Sci. U.S.A.* **119**, e2208090119 (2022).

54. N. H. Sleep, B. F. Windley, Archean plate tectonics: Constraints and inferences. *J. Geol.* **90**, 363–379 (1982).

55. M. Tang, K. Chen, R. L. Rudnick, Archean upper crust transition from mafic to felsic marks the onset of plate tectonics. *Science* **351**, 372–375 (2016).

56. N. D. Greber, N. Dauphas, A. Bekker, M. P. Ptáček, I. N. Bindeman, A. Hofmann, Titanium isotopic evidence for felsic crust and plate tectonics 3.5 billion years ago. *Science* **357**, 1271–1274 (2017).

57. C. Hawkesworth, C. Jaupart, Heat flow constraints on the mafic character of Archean continental crust. *Earth Planet. Sci. Lett.* **571**, 117091 (2021).

58. V. V. Mai, J. Korenaga, What controlled the thickness of continental crust in the Archean? *Geology* **50**, 1091–1095 (2022).

59. T. M. Kusky, Collapse of Archean orogens and the generation of late- to postkinematic granitoids. *Geology* **21**, 925–928 (1993).

60. D. H. Abbott, W. D. Mooney, J. A. VanTongeren, The character of the Moho and lower crust within Archean cratons and the tectonic implications. *Tectonophysics* **609**, 690–705 (2013).

61. J. H. Bédard, A catalytic delamination-driven model for coupled genesis of Archaean crust and sub-continental lithospheric mantle. *Geochim. Cosmochim. Acta* **70**, 1188–1214 (2006).

62. V. Levin, J. A. VanTongeren, A. Servali, How sharp is the sharp Archean Moho? Example from eastern Superior Province. *Geophys. Res. Lett.* **43**, 1928–1933 (2016).

63. D. G. Pearson, J. M. Scott, J. Liu, A. Schaeffer, L. H. Wang, J. van Hunen, K. Szilas, T. Chacko, P. B. Kelemen, Deep continental roots and cratons. *Nature* **596**, 199–210 (2021).

64. Y. Wang, L. Liu, Q. Zhou, Geoid reveals the density structure of cratonic lithosphere. *J. Geophys. Res. Solid Earth* **127**, e2022JB024270 (2022).

65. H. Chen, M. Tang, S. Song, Catastrophic craton destruction via wholesale lithosphere delamination. *Geology* **51**, 460–464 (2023).

66. Y. Wang, Z. Cao, L. Peng, L. Liu, L. Chen, C. Lundstrom, D. Peng, X. Yang, Secular craton evolution due to cyclic deformation of underlying dense mantle lithosphere. *Nat. Geosci.* **16**, 637–645 (2023).

67. H. Kurokawa, J. Foriel, M. Laneuville, C. Houser, T. Usui, Subduction and atmospheric escape of Earth's seawater constrained by hydrogen isotopes. *Earth Planet. Sci. Lett.* **497**, 149–160 (2018).

68. E. C. Pope, D. K. Bird, M. T. Rosing, Isotope composition and volume of Earth's early oceans. *Proc. Natl. Acad. Sci. U.S.A.* **109**, 4371–4376 (2012).

69. J. Dong, R. A. Fischer, L. P. Stixrude, C. R. Lithgow-Bertelloni, Constraining the volume of Earth's early oceans with a temperature-dependent mantle water storage capacity model. *AGU Adv.* **2**, e2020AV000323 (2021).

70. J. H. Bédard, L. B. Harris, P. C. Thurston, The hunting of the snArc. *Precambrian Res.* **229**, 20–48 (2013).

71. J. A. D. Connolly, Computation of phase equilibria by linear programming: A tool for geodynamic modeling and its application to subduction zone decarbonation. *Earth Planet. Sci. Lett.* **236**, 524–541 (2005).

72. T. J. B. Holland, R. Powell, An improved and extended internally consistent thermodynamic dataset for phases of petrological interest, involving a new equation of state for solids. *J. Metamorph. Geol.* **29**, 333–383 (2011).

73. T. J. Holland, E. C. Green, R. Powell, Melting of peridotites through to granites: A simple thermodynamic model in the system KNCFMASHTOCr. *J. Petrol.* **59**, 881–900 (2018).

74. T. Holland, R. Powell, Activity-composition relations for phases in petrological calculations: An asymmetric multicomponent formulation. *Contrib. Mineral. Petrol.* **145**, 492–501 (2003).

75. R. W. White, R. Powell, T. E. Tohnson, The effect of Mn on mineral stability in metapelites revisited: New a-x relations for manganese-bearing minerals. *J. Metamorph. Geol.* **32**, 809–828 (2014).

76. E. C. R. Green, R. W. White, J. F. A. Diener, R. Powell, T. J. B. Holland, R. M. Plain, Activity-composition relations for the calculation of partial melting equilibria in metabasic rocks. *J. Metam. Geol.* **34**, 845–869 (2016).

77. G. Laske, G. Masters, Z. Ma, M. Pasyanos, in *Geophysical Research Abstracts* (EGU General Assembly, 2013), vol. 15, p. 2658.

78. K. Mengel, H. Kern, Evolution of the petrological and seismic Moho-implications for the continental crust-mantle boundary. *Terra Nova* **4**, 109–116 (1992).

79. W. F. McDonough, S.-S. Sun, The composition of the Earth. *Chem. Geol.* **120**, 223–253 (1995).

80. D. G. Pearson, N. Wittig, in *Treatise on Geochemistry (Second Edition),* H. D. Holland, K. K. Turekian, Eds. (Elsevier, 2014), pp. 255–292.

81. J.-F. Moyen, H. Martin, Forty years of TTG research. *Lithos* **148**, 312–336 (2012).

82. J. R. Reimink, A. J. Smye, Subaerial weathering drove stabilization of continents. *Nature* **629**, 609–615 (2024).
